# Differential Requirement for CCR4 and CCR7 during the Development of Innate and Adaptive αβT Cells in the Adult Thymus

**DOI:** 10.4049/jimmunol.1400993

**Published:** 2014-07-02

**Authors:** Jennifer E. Cowan, Nicholas I. McCarthy, Sonia M. Parnell, Andrea J. White, Andrea Bacon, Arnauld Serge, Magali Irla, Peter J. L. Lane, Eric J. Jenkinson, William E. Jenkinson, Graham Anderson

**Affiliations:** * Medical Research Council Centre for Immune Regulation, Institute for Biomedical Research, University of Birmingham, Birmingham B15 2TT, United Kingdom;; † Centre de Recherche en Cancérologie de Marseille, Institut Paoli-Calmettes, INSERM Unité Mixte de Recherche 1068, Centre National de la Recherche Scientifique Unité Mixte de Recherche 7258, Aix-Marseille University, UM 105, F-13009 Marseille, France; and; ‡ Centre d’Immunologie de Marseille-Luminy, INSERM Unité Mixte de Recherche 631, Centre National de la Recherche Scientifique Unité Mixte de Recherche 6102, Aix-Marseille University, UM 2, F-13009 Marseille, France

## Abstract

αβT cell development depends upon serial migration of thymocyte precursors through cortical and medullary microenvironments, enabling specialized stromal cells to provide important signals at specific stages of their development. Although conventional αβT cells are subject to clonal deletion in the medulla, entry into the thymus medulla also fosters αβT cell differentiation. For example, during postnatal periods, the medulla is involved in the intrathymic generation of multiple αβT cell lineages, notably the induction of Foxp3^+^ regulatory T cell development and the completion of invariant NKT cell development. Although migration of conventional αβT cells to the medulla is mediated by the chemokine receptor CCR7, how other T cell subsets gain access to medullary areas during their normal development is not clear. In this study, we show that combining a panel of thymocyte maturation markers with cell surface analysis of CCR7 and CCR4 identifies distinct stages in the development of multiple αβT cell lineages in the thymus. Although Aire regulates expression of the CCR4 ligands CCL17 and CCL22, we show that CCR4 is dispensable for thymocyte migration and development in the adult thymus, demonstrating defective T cell development in *Aire^−/−^* mice is not because of a loss of CCR4-mediated migration. Moreover, we reveal that CCR7 controls the development of invariant NKT cells by enabling their access to IL-15 *trans*-presentation in the thymic medulla and influences the balance of early and late intrathymic stages of Foxp3^+^ regulatory T cell development. Collectively, our data identify novel roles for CCR7 during intrathymic T cell development, highlighting its importance in enabling multiple αβT cell lineages to access the thymic medulla.

## Introduction

In the thymus, the development of T cells bearing the αβTCR involves the progressive migration of immature thymocyte precursors through distinct intrathymic microenvironments formed from specialized thymic stromal cells (1, 2). Although immature CD4^−^8^−^ thymocytes are localized within subcapsular regions of the thymus, cortical regions house CD4^+^8^+^ thymocytes expressing low levels of αβTCR that represent precursors of conventional, natural Foxp3^+^ regulatory T cells (Foxp3^+^ nT-Reg) and CD1d-restricted invariant NKT (iNKT). In contrast, medullary areas contain more mature CD4^+^ and CD8^+^ single-positive (SP) cells that subsequently emigrate from the thymus to populate peripheral tissues (2). Importantly, the thymus medulla and medullary thymic epithelial cells (mTEC) in particular play important functional roles during the intrathymic development of multiple αβT cell subsets. For example, although several studies have highlighted the important role played by mTEC in tolerance induction via the clonal deletion of autoreactive conventional αβT cells (3, 4), the medulla also plays a key role in generating αβT cell subsets with key regulatory functions in the immune system (5). Importantly, Foxp3^+^ nT-Reg development has been shown to be a multistage process (6, 7), with Foxp3^−^CD25^+^ nT-Reg precursors in particular requiring interactions with mTEC for their generation (8). In addition, we recently showed that mTEC play an important role during iNKT cell development in the thymus by providing IL-15 *trans*-presentation to expand newly generated iNKT cells following their CD1d-dependent positive selection in the cortex (9). Collectively, such observations indicate that a key shared step in the intrathymic development of multiple αβT cell lineages is the migration of thymocytes from the cortex to the medulla.

Many studies have highlighted the importance of chemokines and their receptors during the intrathymic migration that occurs during conventional αβT cell development. In particular, CCR7 upregulation occurs during positive selection (10, 11) and plays a key role in tolerance induction, a finding compatible with its role in controlling thymocyte access to medullary microenvironments and its effects on TCR signaling (10, 12). The role of CCR7 in cortex to medulla migration of SP thymocytes fits well with expression of both CCR7 ligands CCL19 and CCL21 in the medulla (13, 14). Interestingly, a recent study also demonstrated a role for CCR7 in the medullary localization and thymic development of γδT cells (15), suggesting that it can influence multiple T cell lineages. Indeed, Foxp3^+^ thymocytes have been reported to accumulate in the cortex of *Ccr7^−/−^* mice (16, 17), although the impact of CCR7 deficiency on distinct nT-Reg progenitors and more mature Foxp3^+^ nT-Reg stages has not been fully addressed. Moreover, the chemokine receptors controlling the intrathymic migration of iNKT cells, enabling them to access the thymus medulla during their normal development, are not clear. Although CCR7 deficiency does not totally eliminate SP thymocytes from thymic medullary regions (10, 12), pertussis toxin treatment has a more profound effect (18, 19), thereby implicating other chemokines receptors in cortex to medulla migration. In line with this, positive selection is known to alter the in vitro responsiveness of thymocytes to several chemokines including CCL17 and CCL22 (20), representing ligands for CCR4 (21). Moreover, Aire expression by MHC class II^high^ mTEC is known to influence intrathymic chemokine production (22, 23), including the ligands for CCR4 (23). Indeed, impaired CCR4-mediated thymocyte migration recently has been suggested (24) to help explain defects in the development of both conventional and Foxp3^+^ nT-Reg that are linked to the autoimmunity seen in *Aire^−/−^* mice (22, 25). However, although CCR4 has been studied in the peripheral immune system, notably in the context of skin-homing of T cells (26), its role during the development of distinct αβT cell lineages in the adult thymus, either individually or in combination with CCR7, has not been studied.

In this study, we show that combined cell surface expression of CCR4 and CCR7 can be used to highlight multiple developmental stages of conventional Foxp3^+^ nT-Reg and iNKT cell lineages in the thymus. Notably, CCR7 marks early iNKT cell subsets, whereas CCR4 identifies a narrow window during the early stages of positive selection of both conventional and regulatory SP4 T cells, prior to their CCR7 expression. In addition, through analysis of single-knockout *Ccr4^−/−^* and *Ccr7^−/−^* mice and the generation of *Ccr4^−/−^*x*Ccr7^−/−^* double-knockout (DKO) mice, we show that in the adult thymus, CCR4 is dispensable for thymocyte maturation, even in the context of CCR7 deficiency. Such findings argue against intrathymic redundancy of these chemokine receptors and demonstrate that Aire-mediated control of CCL17/CCL22 expression does not underlie the defective αβT cell development seen in adult *Aire^−/−^* mice (27). Moreover, we reveal previously unreported roles for CCR7 in the development of T cell lineages that arise postnatally. Thus, CCR7 is required both in the intrathymic development of iNKT cells by controlling access to mTEC-derived IL-15 and in control of the intrathymic balance of Foxp3^+^CD25^+^ nT-Reg and their Foxp3^−^CD25^+^ precursors. Such observations collectively demonstrate new roles for CCR7 during the intrathymic development of mTEC-dependent αβT cell subsets.

## Materials and Methods

### Mice

Wild-type (WT) CD45.2^+^ C57BL/6, congenic CD45.1^+^ C57BL/6 (BoyJ), Rag2GFP (28), C57BL/6 Foxp3GFP reporter mice (29), *Ccr4*^−/−^ (30), *Ccr7^−/−^* (31), *Ccr4^−/−^*x*Ccr7^−/−^*, and *Zap 70^−/−^* (32) were bred at the University of Birmingham in accordance with Home Office Regulations. Adult mice were used at 8–12 wk of age. Embryonic mice were generated by timed pregnancies and vaginal plug detection was designated day 0. All animal experiments were performed in accordance with University of Birmingham (Local Ethical Review Panel) and national United Kingdom Home Office regulations.

### Abs, flow cytometry, and cell sorting

Thymocyte suspensions were stained with the following Abs: PECy7/PE/Alexa Fluor 700 anti-CD4 (clone GK1.5; eBioscience) or PerCP-Cy5.5/allophycocyanin eFluor780/V500 anti-CD4 (clone RM4-5; eBioscience/BD Biosciences), eFluor450/FITC/V500/PE anti-CD8 clone 53-6.7 (eBioscience/BD Biosciences) or biotinylated anti-CD8 clone (YTS156.7.7; BioLegend), allophycocyanin eFluor780 anti-TCRβ (clone H57-597; eBioscience), PE anti-CD3ε (clone 145-2C11; eBioscience), FITC/PerCP-Cy5.5 anti-CD69 (clone H1.2F3; eBioscience), allophycocyanin anti-CD62L (clone MEL-14; BioLegend), FITC/Alexa Fluor 700 anti-CD44 (clone IM7; eBioscience), allophycocyanin eFluor780/PE anti-HSA/CD24 (clone M1/69; BD Biosciences/eBioscience), biotinylated/FITC anti-Qa2 (clone 695H1-9.9; BioLegend/eBioscience), eFluor780/eFluor450 anti-CD45.1 (clone A20; eBioscience), PE/Alexa Fluor 700 anti-CD45.2 (clone 104; eBioscience), allophycocyanin/PE anti-CD25 (clone PC61/PC61.5; BioLegend/eBioscience), FITC anti-NK1.1 (clone PK136; eBioscience), allophycocyanin/biotinylated anti-CCR4 (clone 2G12; BioLegend), and Ki67 (SolA15; eBioscience). To detect cell surface CCR7 expression, thymocytes were incubated in recombinant CCL19-Ig (eBioscience), followed by biotinylated goat anti-human Ig (eBioscience). All biotinylated Abs were revealed with PECy7-conjugated streptavidin (eBioscience). Brilliant Violet 421/allophycocyanin-conjugated CD1d tetramers loaded with PBS57 to detect iNKT cells were obtained from the National Institutes of Health Tetramer Facility. For intracellular staining of Foxp3, cells were fixed and permeabilized using the Foxp3/Transcription Factor Staining Buffer Set (eBioscience), according to the manufacturer’s protocol and stained with PE anti-Foxp3 (clone FJK-16s; eBioscience). Abs used to identify and isolate TEC populations were as follows: anti-CD45 allophycocyanin Cy7/allophycocyanin eFluor 780 (30-F11), anti-EpCAM1 allophycocyanin (G8.8), anti-Ly51 PE (6C3), anti-CD80 Brilliant Violet 421 (16-10A1; BioLegend), MHC class II FITC (AF6-1201; BD Pharmingen). All flow cytometry was performed on a BD Fortessa Analyzer using FACSDiva6.2 software (BD Biosciences), with data subsequently analyzed with FlowJo 8.7 software (Tree Star). Purified populations of thymocytes and TEC were sorted from seven day fetal thymus organ cultures using a Mo-Flo XDP cell sorter, as described previously (33).

### Immunohistochemistry

Adult thymus tissues from host mice were frozen, 7-μm sections were cut, and then fixed in acetone and stained with the following Abs: the mTEC marker ERTR5 (a gift from Dr. W. van Ewijk, RIKEN Research Center for Allergy and Immunology, Yokohama, Japan), detected with Alexa Fluor 594 goat anti-rat IgM (Invitrogen), biotinylated anti-CD45.1 (clone A20; eBioscience), detected by Streptavidin Alexa 647 (Invitrogen), FITC anti-CD45.2 (clone 104; eBioscience), followed by rabbit anti-FITC (Invitrogen), and donkey anti-rabbit 488 (Invitrogen), biotinylated anti-CD8 (clone YTS156.7.7; BioLegend), detected by Streptavidin Alexa 488 (Invitrogen), and purified anti-CD4 (clone GK 1.5; eBioscience), conjugated to Alexa 647 (Invitrogen). Images were obtained using a LSM 780 microscope and analyzed using LSM software. (Zeiss). For confocal quantitation, a minimum of three separate thymus sections at least 10 sections apart were analyzed for each mouse. Calculation of cell frequency within a defined area was performed as previously described (34), where square fields of 100 × 100μm were arbitrarily set within either medullary or cortical regions, at least 100 μm from the CMJ.

### Bone marrow chimeras

To investigate the role of CCR4 in T cell development in a competitive setting, mixed WT/WT and *Ccr4^−/−^*/WT chimaeras were established in a manner similar to that used to analyze the role of CCR7 (10). In brief, bone marrow cells were obtained from the femurs and tibias of *Ccr4^−/−^* (CD45.2), C57BL/6 (CD45.2), or Boy J (CD45.1) mice. Bone marrow was T cell depleted using PE-labeled anti-CD3, anti-CD4, and anti-CD8 Abs and anti-PE microbeads (Miltenyi Biotec). CD45.1^+^ hosts were lethally irradiated (two split doses of 450 rad) and reconstituted 24 h later with 5 × 10^6^ T cell–depleted bone marrow cells from either CD45.2^+^ donors (either WT C57BL/6 or *Ccr4^−/−^*) or CD45.1^+^ (WT C57BL/6 Boy J) donors. Mice were analyzed 5–6 wk postreconstitution.

### IL-15/IL-15Rα complex administration

Soluble IL-15/IL-15Rα complexes were prepared as described previously (9, 35). A total of 2.5 mg rIL-15 (PeproTech) was mixed with 15 mg IL-15Rα (R&D Systems) in 50 μl PBS and incubated for 30 min at 37°C and then made up to a total volume of 300 μl with PBS prior to i.p. injection into WT and *Ccr7^−/−^* mice. Mice were sacrificed 4 d postinjection, and thymic samples were analyzed for iNKT cell populations by flow cytometry.

### Quantitative PCR

Quantitative PCR (qPCR) analysis of freshly sorted thymocyte populations was performed exactly as described (33). Primers used were as follows: β-actin (QuantiTect Mm *Actb* 1SG Primer Assay Qiagen QT00095242); *Foxo1*, forward, 5′-TGTCAGGCTAAGAGTTAGTGAGCA-3′, and reverse, 5′-GGGTGAAGGGCATCTTTG-3′; *Klf2*, forward, 5′-CTCAGCGAGCCTATCTTGCC-3′, and reverse, 5′-CACGTTGTTTAGGTCCTCATCC-3′; *S1pr1*, forward, 5′-AAATGCCCCAACGGAGACT-3′, and reverse, 5′-CTGATTTGCTGCGGCTAAATTC-3′; *Ccl17*, forward, 5′-AGTGGAGTGTTCCAGGGATG-3′, and reverse, 5′-CCAATCTGATGGCCTTCTTC-3′; and *Ccl22*, forward, 5′-CTGATGCAGGTCCCTATGGT-3′, and reverse, 5′-GGAGTAGCTTCTTCACCCAG-3′.

## Results

### CCR4 and CCR7 combine to identify serial stages in the development of both conventional and regulatory αβT cells

**FIGURE 1.**
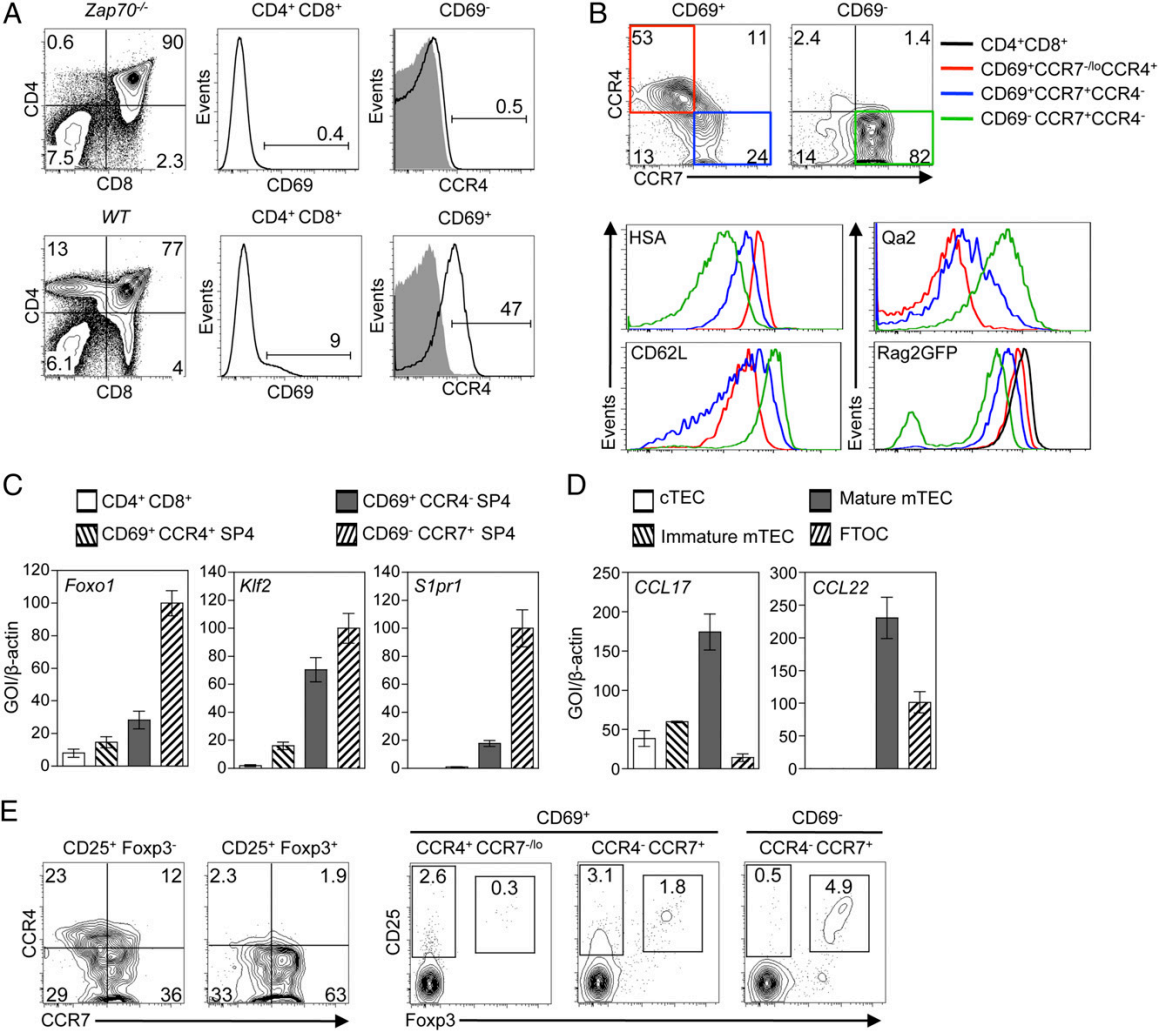
CCR4 and CCR7 define distinct stages of conventional and Foxp3^+^ regulatory αβT cell development. (**A**) Adult CD4^+^8^+^69^−^ thymocytes from *Zap70^−/−^* mice (*upper panels*), representing cells at a preselection stage of development, and CD4^+^8^+^69^+^ thymocytes from WT mice (*lower panels*), representing cells undergoing selection. These populations were analyzed for expression of CD4, CD8, CD69, and CCR4 by flow cytometry. (**B**) CD69^+^ and CD69^−^ subsets of CD4^+^8^−^αβTCR^hi^ (SP4) adult thymocytes from Rag2GFP mice analyzed for expression of CCR4 and CCR7, together with HSA, Qa2, CD62L, and Rag2GFP levels. Colored boxes in the *upper panels* relate to colored lines in the histograms shown, and levels of Rag2GFP in CD4^+^8^+^ thymocytes (black line) is shown for comparison. (**C**) qPCR analysis of mRNA levels for the indicated genes in CD4^+^8^+^ thymocytes and CD69/CCR4/CCR7 SP4 adult thymocyte subsets. (**D**) qPCR analysis of *Ccl17*/*Ccl22* mRNA expression in the indicated TEC subsets. In all cases, mRNA levels were normalized to β-actin; fold levels represent the mean (± SEM) of replicate reactions, and data shown represent at least two independent experiments. (**E**) *Left panels*, CCR4/CCR7 expression within CD25^+^Foxp3^−^ nT-Reg precursors and CD25^+^Foxp3^+^ nT-Reg subsets of TCRβ^hi^ SP4 thymocytes; *right panels*, CD25 and Foxp3 in TCRβ^hi^ SP4 adult thymocyte subsets identified on the basis of CD69, CCR4, and CCR7 expression. All flow cytometric analysis is typical of three experiments.

To identify chemokine receptors expressed at distinct stages of positive selection that may play a role in the recruitment of developing thymocytes to medullary thymic regions, we performed a flow cytometric screen of chemokine receptor expression, coupled with analysis of chemokine mRNA expression in purified TEC compartments. Specifically, to identify chemokine receptors induced by TCR-mediated positive selection, we compared CD4^+^8^+^69^+^ thymocytes from WT mice, representing cells undergoing selection, with CD4^+^8^+^69^−^ thymocytes from *Zap70^−/−^* mice, which are blocked at a preselection stage of thymocyte development (32). Of the molecules analyzed and consistent with in vitro thymocyte migration data and *Ccr4* mRNA analysis (20), we found that CD4^+^8^+^CD69^+^ thymocytes undergoing thymic selection, but not preselection CD4^+^8^+^CD69^-^ cells, expressed readily detectable cell surface expression of CCR4 (Fig. 1A). Interestingly, analysis of CCR4 in combination with CCR7 within CD69^+^ SP4 thymocytes identified CCR4^+^CCR7^−/lo^ and CCR4^−^CCR7^+^ subsets, whereas CD69^−^ SP4 thymocytes were predominantly CCR4^−^CCR7^+^ (Fig. 1B). In contrast to SP4 thymocytes, αβTCR^+^ SP8 thymocytes lacked CCR4 expression (data not shown). Moreover, analysis with additional markers including CD24, CD62L, Qa2, and notably levels of Rag2GFP (a direct indicator of thymocyte maturational status) (36) indicated the following developmental sequence within SP4 thymocytes: CD69^+^CCR4^+^CCR7^−/lo^, CD69^+^CCR4^−^CCR7^+^, and then CD69^-^CCR4^−^CCR7^+^ (Fig. 1B). Such a sequence is compatible with qPCR analysis of mRNAs linked to later stage thymocyte migration and thymus egress, including *Foxo1*, *Klf2*, and *S1pr1,* which were notably absent from CCR4^+^ thymocytes (Fig. 1C). Thus, CCR4 expression identifies a narrow window of thymocyte positive selection, with SP4 thymocytes expressing CCR4 prior to their acquisition of high levels of CCR7. In line with the pattern of CCR4 expression and consistent with a potential role during cortex to medulla migration, analysis of defined TEC compartments showed that CD80^+^ mTEC expressed the highest levels of *Ccl17* and *Ccl22* mRNA (Fig. 1D).

Similar to conventional αβT cell development, Foxp3^+^ nT-Reg development in the thymus can be subdivided into distinct stages (6, 7). As we recently showed that the generation of CD25^+^Foxp3^−^ nT-Reg precursors is dependent on contact with mTEC (8), we next examined the expression of chemokine receptors at distinct stages in Foxp3^+^ nT-Reg development that might play a role in their migration to medullary microenvironments. Direct gating on CD25^+^Foxp3^+^ SP4 nT-Reg and their CD25^+^Foxp3^−^ SP4 precursors showed that although the former lacked CCR4 expression, CD25^+^Foxp3^−^ nT-Reg precursors contained subsets of CCR4^+^ and CCR7^+^ cells (Fig. 1E, *left panels*). Further analysis showed that CD25^+^Foxp3^−^ nT-Reg precursors were primarily contained within CD69^+^CCR4^+^CCR7^−/lo^ and CD69^+^CCR4^−^CCR7^+^ SP4 thymocyte subsets (Fig. 1E, *right panels*). In contrast, although CD25^+^Foxp3^+^ SP4 nT-Reg cells were largely absent from the CD69^+^CCR4^+^CCR7^−/lo^ population, they first emerged in the CD69^+^CCR4^−^CCR7^+^ subset and were most abundant within CD69^−^CCR4^−^CCR7^+^ cells. Thus, CCR4 is expressed during early stages in the development of both conventional and regulatory SP4 T cell lineages in the thymus.

### CCR4 is dispensable for both conventional and Foxp3^+^ regulatory T cell development in the adult thymus

**FIGURE 2.**
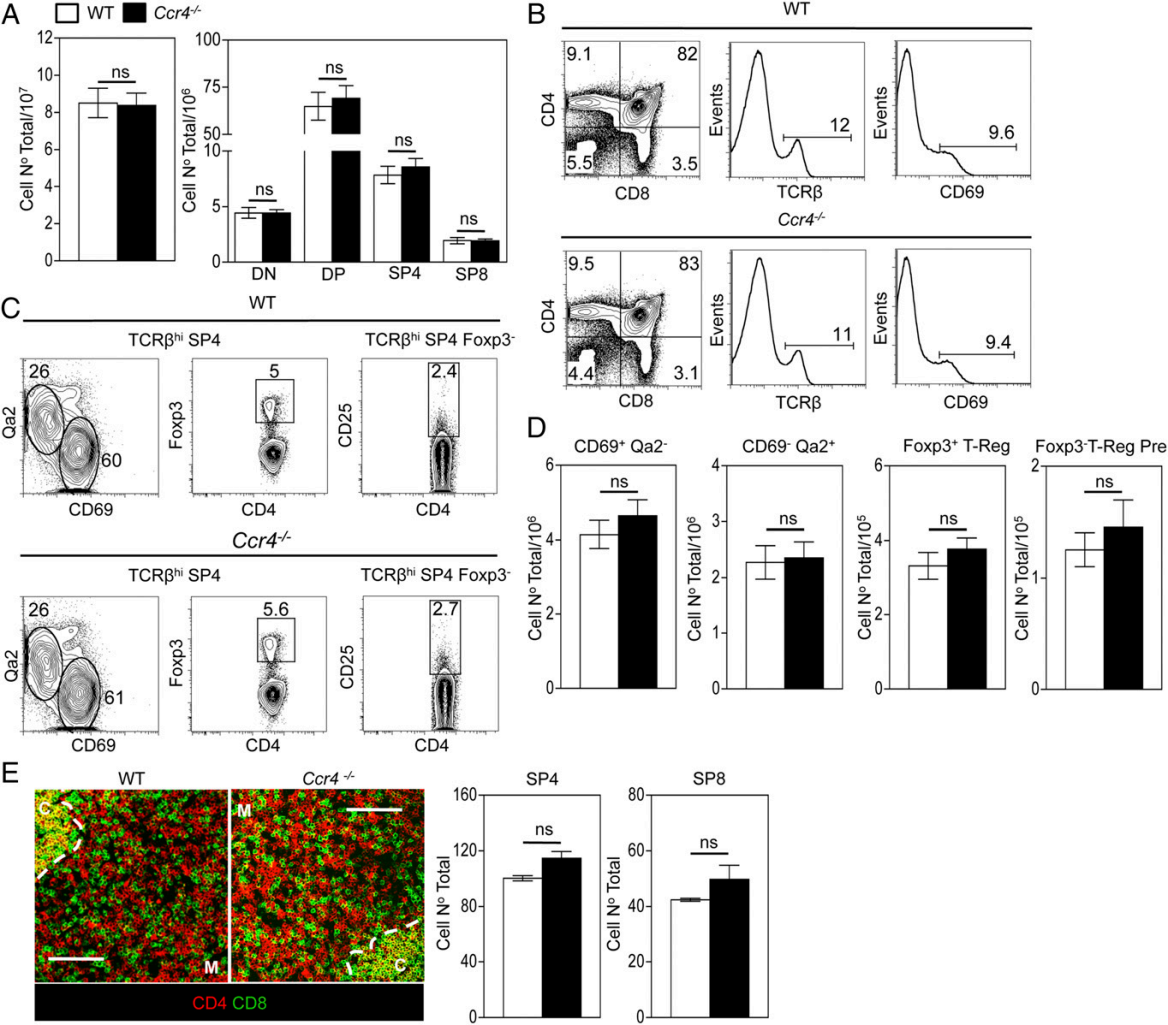
CCR4 is dispensable for both conventional and Foxp3^+^ regulatory αβT cell development in the adult thymus. (**A**) Absolute numbers of total and CD4/CD8 thymocyte subsets obtained from WT (□) and *Ccr4^−/−^* (▪) mice. (**B**) Representative flow cytometric analysis of CD4, CD8, CD69, and TCRβ expression on total thymocytes from WT (*upper*) and *Ccr4^−/−^* (*lower*) adult mice. (**C**) Representative flow cytometric analysis of CD69/Qa2 or Foxp3 expression on TCRβ^hi^ SP4 WT (*upper panels*) and *Ccr4^−/−^* (*lower panels*) thymocytes and CD25 expression on Foxp3^−^TCRβ^hi^ SP4 cells. (**D**) The absolute numbers of the indicated SP4 thymocyte subsets from WT (□) and *Ccr4^−/−^* (▪) mice. (**E**) Representative confocal analysis of thymus sections from WT and *Ccr4^−/−^* mice stained for expression of CD4 (red) and CD8 (green), where C denotes cortex, M denotes medulla, and the dotted line denotes the corticomedullary junction (CMJ). Scale bars, 100 μm. Images were used to quantitate the frequency of medullary resident SP4 and SP8 thymocytes in WT (□) and *Ccr4^−/−^* (▪) thymic sections. (D and E) Error bars represent SEM using an unpaired Student two-tailed *t* test. Data in (A)–(D) are representative of at least six mice per group in a minimum of two experiments, and data in (E) are representative of three mice per group.

**FIGURE 3.**
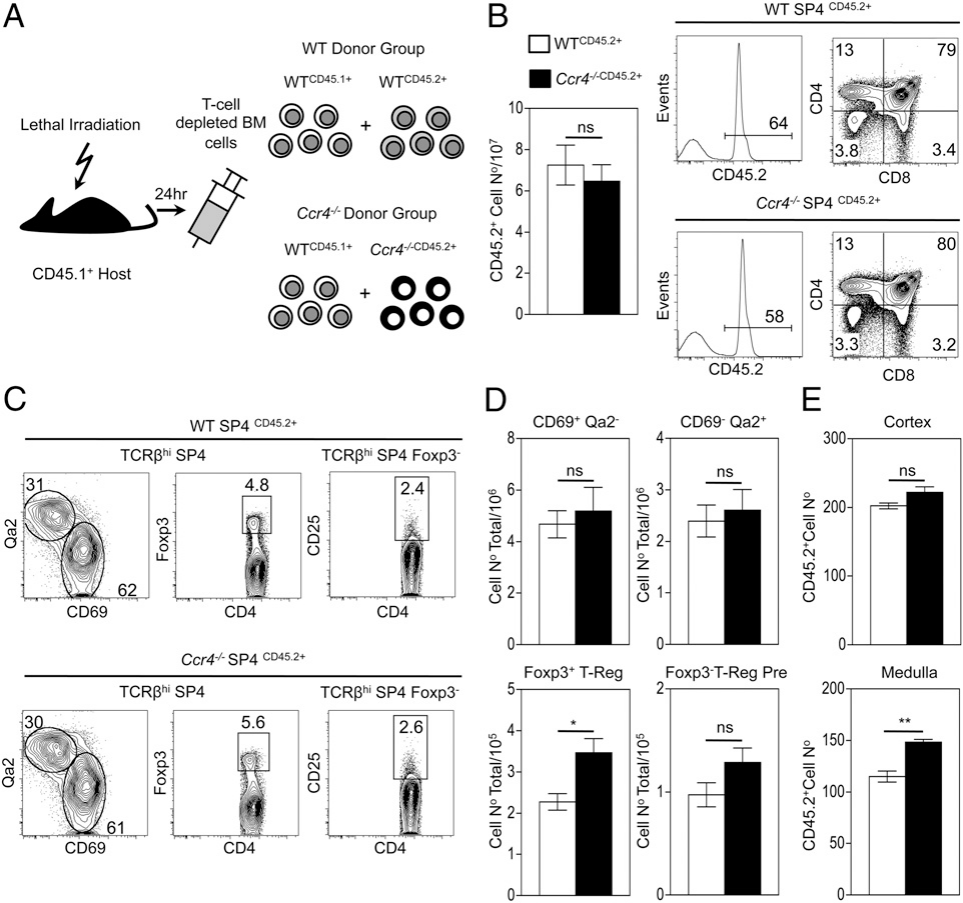
Foxp3^+^ regulatory and conventional αβT cell development in competitive WT:*Ccr4^−/−^* mixed bone marrow chimaeras. (**A**) A summary of the experimental strategy used to construct WT CD45.1/WT CD45.2 and WT CD45.1/*Ccr4^−/−^* CD45.2 mixed bone marrow chimaeras. (**B**) The number of CD45.2^+^ WT (□) and CD45.2^+^*Ccr4^−/−^* (▪) thymocytes recovered from such mice, taken together with representative flow cytometric analysis of thymocyte expression of CD45.2, CD4, and CD8. (**C**) Representative flow cytometric analysis of WT CD45.2^+^ (*upper panels*) and *Ccr4^−/−^*CD45.2^+^ (*lower panels*) TCRβ^hi^ SP4 thymocytes for CD69/Qa2 and Foxp3 expression, along with CD25 expression by Foxp3^−^TCRβ^hi^ SP4 cells. (**D**) Absolute numbers of the indicated WT (□) and *Ccr4^−/−^* (▪) SP4 thymocyte populations. (**E**) Confocal quantitation of the frequency of CD45.2^+^ WT (□) and CD45.2^+^*Ccr4^−/−^* (▪) thymocytes within cortex (*upper panel*) and medulla (*lower panel*) regions of sections of chimaera thymuses. (B, D, and E) Error bars represent SEM. Data in (B)–(D) represents at least six mice per group in a minimum of two experiments; data in (E) represents three mice per group. An unpaired Student two-tailed *t* test was used: *p < 0.05, **p < 0.01.

Unlike *Ccr7^−/−^* mice, detailed analysis of intrathymic migration and T cell development in adult *Ccr4^−/−^* mice (30) has not been performed. Although CCR4 expression mapped to a narrow developmental window in thymic positive selection, analysis of thymocyte development in adult *Ccr4^−/−^* mice revealed no major alterations in conventional SP4 and Foxp3^+^ regulatory αβT cell development. This included normal proportions and numbers of CD69^+^Qa2^low^,CD69^−^Qa2^high^ conventional SP4 αβT cell stages (Fig. 2A–D), as well as CD25^+^Foxp3^−^ nT-Reg precursors and CD25^+^Foxp3^+^ nT-Reg (Fig. 2A–D). Furthermore, confocal quantitation of the intrathymic positioning of CD4^+^ and CD8^+^ SP thymocytes in adult thymus showed a comparable density of these cells within medullary regions of WT and *Ccr4^−/−^* thymuses (Fig. 2E), arguing against a role for CCR4 in cortex-to-medulla migration of positively selected thymocytes. Moreover, in an earlier study analyzing mice at E18 of gestation, we found no overt differences between *Ccr4^−/−^* and WT embryonic thymus lobes, in terms of total thymocyte numbers, cortical and medullary architecture and proportions of immature and mature thymocytes (Ref. 37; data not shown). Thus, initial analysis suggests that absence of CCR4 during development does not have a major impact on fetal thymus populations. As the impact of CCR7 deficiency on developing thymocytes is exacerbated in the presence of WT competitors (10), we performed similar experiments to assess the impact of CCR4 deficiency in a competitive setting. T cell–depleted mixed (50:50) bone marrow chimaeras between WT (CD45.1^+^):*Ccr4^−/−^* (CD45.2^+^), or WT (CD45.1^+^):*Ccr4^−/−^* (CD45.2^+^) partners were generated as previously described (10) and harvested after 5 wk (experimental design illustrated in Fig. 3A). The capacity of *Ccr4^−/−^* thymocytes to undergo a normal program of both conventional and Foxp3^+^ regulatory (Fig. 3B–D) αβT cell development was not altered in the presence of competition with WT thymocytes, whereas the ability of *Ccr4^−/−^* thymocytes to gain access to medullary thymic areas was comparable to that of WT competitors (Fig. 3E). Thus, no defects in *Ccr4^−/−^* thymocyte migration and development were revealed by the presence of competition from WT counterparts.

**FIGURE 4.**
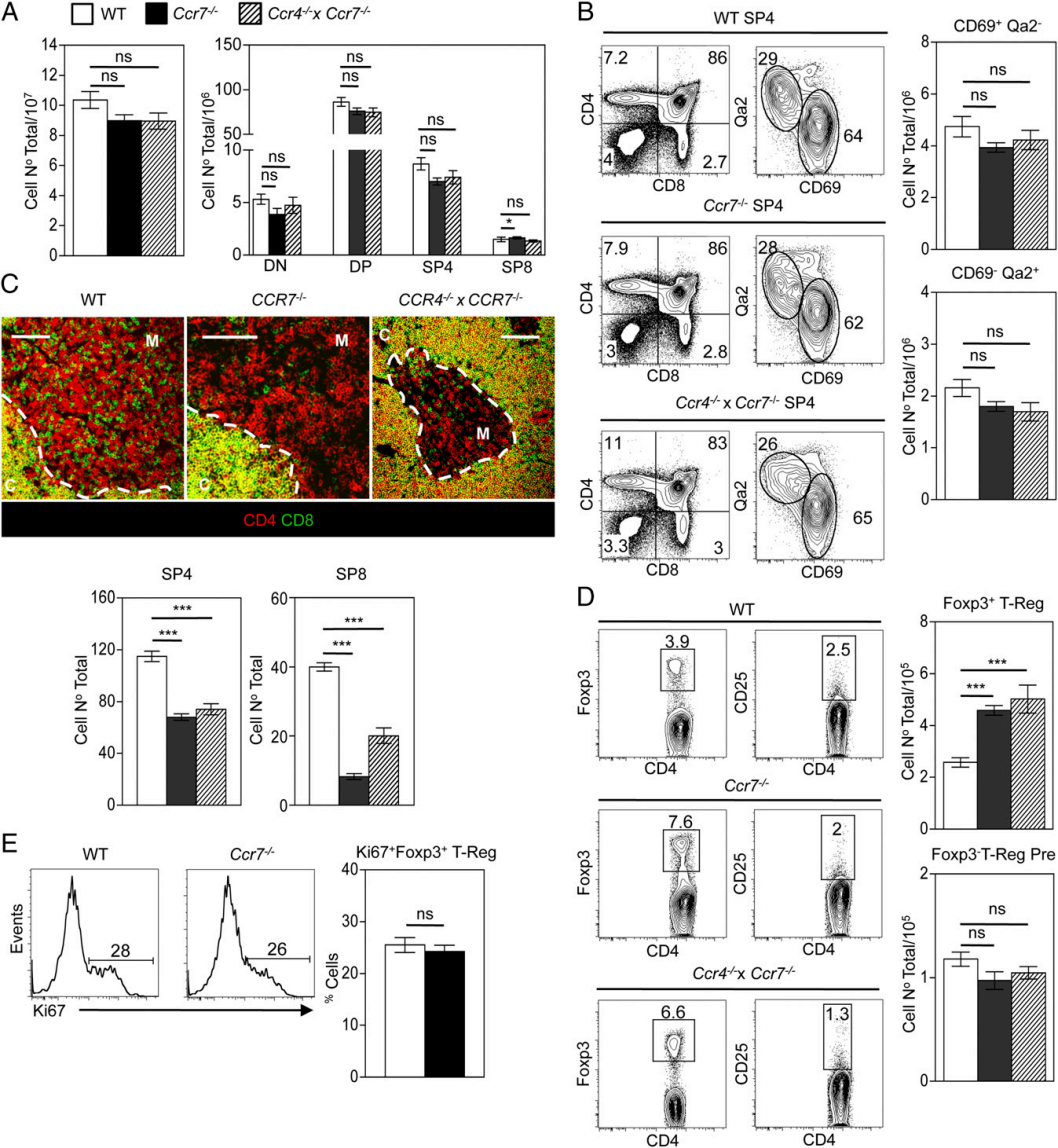
CCR7 controls the intrathymic balance of Foxp3^+^ regulatory T cells. (**A**–**D**) Analysis of adult thymocytes obtained from WT (open bars), *Ccr7^−/−^* (black bars) and *Ccr4^−/−^*x*Ccr7^−/−^* (shaded bars) mice. (A) Total and CD4/CD8 subset thymus cellularity obtained from the indicated mice. (B) Representative flow cytometric analysis of CD69/Qa2 expression on TCRβ^hi^ SP4 thymocytes from WT (*top panels*), *Ccr7^−/−^* (*middle panels*), and *Ccr4^−/−^*x*Ccr7^−/−^* (*bottom panels*) and the absolute numbers of the indicated populations. (C) Representative confocal analysis of adult thymus sections, stained for expression of CD4 (as detected with Alexa 647; red) and CD8 (as detected with Alexa 488; green), where C denotes cortex, M denotes medulla, the dotted line denotes the corticomedullary junction (CMJ); scale bars, 100 μm. Images were used to quantitate the frequency of medullary resident SP4 and SP8 thymocytes in WT, *Ccr7^−/−^*, and *Ccr4^−/−^*x*Ccr7^−/−^* mice. Dot plots in the *left panels* (D) show expression of Foxp3 in TCRβ^hi^ SP4 thymocytes from WT (*top panels*), *Ccr7^−/−^* (*middle panels*), and *Ccr4^−/−^*x*Ccr7^−/−^* (*bottom panels*) mice, whereas dot plots in the *right panels* (D) show CD25 expression within the Foxp3^−^ fraction of TCRβ^hi^ SP4 thymocytes, along with the absolute numbers of the indicated populations. (**E**) Representative flow cytometric analysis and percentages for the proliferation marker Ki67 in CD25^+^Foxp3^+^TCRβ^hi^ SP4 nT-Reg from WT and *Ccr7^−/−^* mice, where Ki67^+^ gating was set using an isotype control. Data in (A), (B), and (D) represent at least six mice per group in a minimum of two experiments; data in (C) and (E) represent a minimum of three mice per group. (A–D) Error bars represent SEM; an unpaired Student two-tailed *t* test was used: ***p < 0.001.

Because the data above indicate that CCR4 and CCR7 are expressed at similar stages of intrathymic T cell development, we investigated whether compensatory mechanisms involving these two chemokine receptors could be playing a role during the intrathymic migration and development of αβT-cells. To this end, we generated *Ccr4^−/−^*x*Ccr7^−/−^* DKO mice. Adult thymuses from WT, *Ccr7^−/−^* single knockout, and *Ccr4^−/−^*x*Ccr7^−/−^* DKO thymuses showed comparable overall thymocyte cellularity (Fig. 4A) and proportions and numbers of conventional αβT cell subsets, including immature (CD69^+^Qa2^low^) and mature (CD69^−^Qa2^hi^) subsets of SP4 thymocytes (Fig. 4B). Although quantitation of SP thymocyte positioning by confocal analysis of frozen tissue sections confirmed a defect in medullary accumulation of *Ccr7^−/−^* SP cells (10), no additive effect was observed in adult *Ccr4^−/−^*x*Ccr7^−/−^* DKO mice (Fig. 4C). Collectively, our findings show that CCR4-mediated migration does not explain the residual migration of SP thymocytes to the medullary areas seen in adult *Ccr7^−/−^* mice and that, in the context of the steady-state adult thymus, αβT cell development occurs normally in the absence of CCR4.

### A role for CCR7 during the intrathymic maturation of iNKT and Foxp3^+^ regulatory T cells

Our previous studies have shown that the generation of both Foxp3^+^ nT-Reg and CD1d-restricted iNKT-cells depends on normal thymus medulla development and interactions with mTEC (8, 9). Given the role of CCR7 during thymocyte migration, we next analyzed the intrathymic generation of these lineages in mice lacking CCR7, either individually or in combination with a lack of CCR4. In contrast to that seen with normal conventional αβT-cells (Fig. 4A, 4B), we found alterations in the CD25^+^Foxp3^+^ nT-Reg lineage in *Ccr7^−/−^* mice, which were also evident to a similar degree in *Ccr4^−/−^*x*Ccr7^−/−^* DKO mice. Thus, although both CCR4 and CCR7 were expressed by CD25^+^Foxp3^−^ SP4 nT-Reg progenitors (Fig. 1E), their frequency in both *Ccr7^−/−^* and *Ccr4^−/−^*x*Ccr7^−/−^* DKO mice remained unaltered (Fig. 4D). In contrast, the more mature CD25^+^Foxp3^+^ nT-Reg subset showed an increase in the thymuses of *Ccr7^−/−^* mice that was evident to a comparable extent in *Ccr4^−/−^*x*Ccr7^−/−^* DKO mice (Fig. 4D), findings consistent with their expression of CCR7 but not CCR4 (Fig. 1E). Although the reasons for the increase are not fully understood, analysis of the proliferation marker Ki67 by CD25^+^Foxp3^+^ nT-Reg in WT and *Ccr7^−/−^* mice (Fig. 4E) argues against the idea that it is reflective of increased nT-Reg proliferation. Thus, CCR7 plays a role in controlling the size of the intrathymic CD25^+^Foxp3^+^ nT-Reg compartment but not at the level of generation of their CD25^+^Foxp3^−^ nT-Reg progenitors.

**FIGURE 5.**
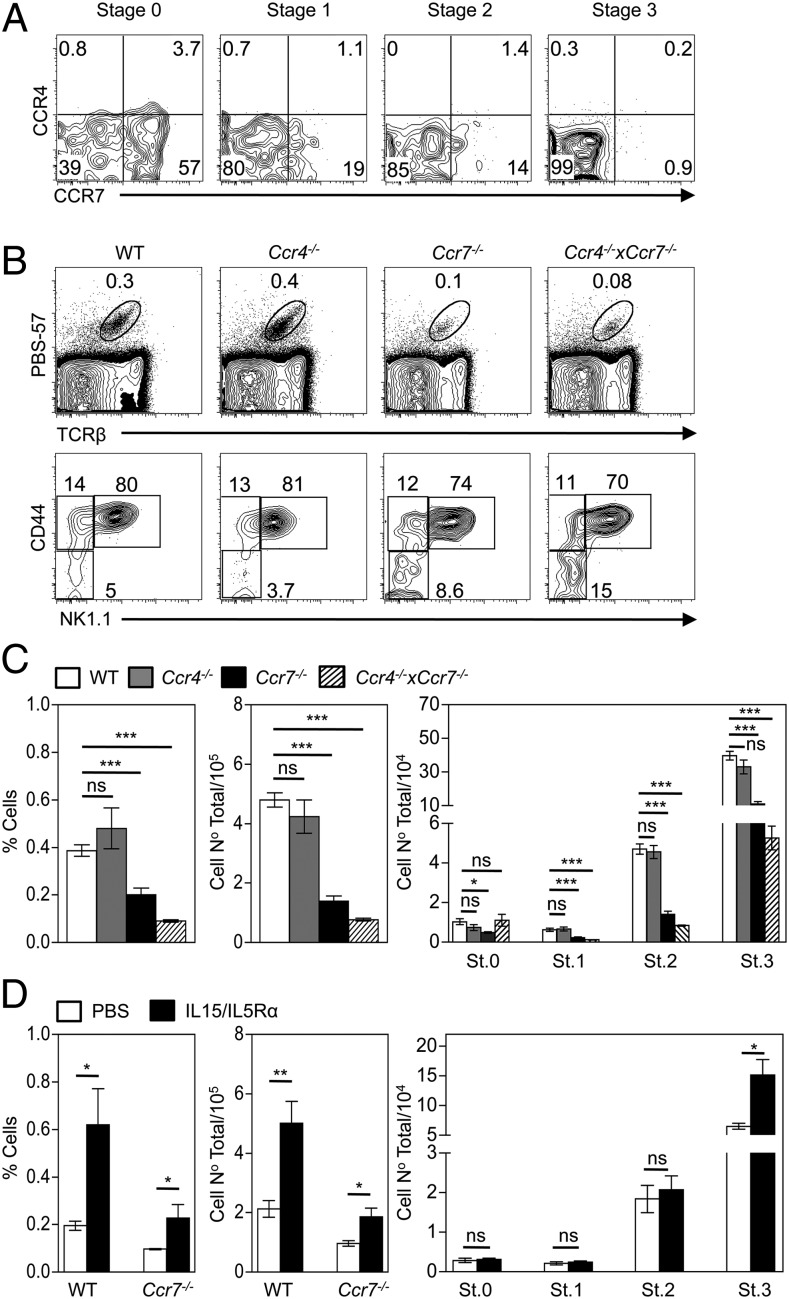
iNKT cell development in the thymus is CCR7 dependent. (**A**) CCR4/CCR7 expression during PBS57^+^ iNKT cell development in WT mice, where CD24/CD44/NK1.1 were used to identify the following stages: stage (St) 0 (CD24^+^CD44^−^NK1.1^−^), St.1 (CD24^−^CD44^−^NK1.1^−^), St.2 (CD44^+^NK1.1^−^), and St.3 (CD44^+^NK1.1^+^). (**B**) Representative flow cytometric analysis of total iNKT cells (*upper panels*) and their distinct developmental stages (*lower panels*); (**C**) the percentage and absolute numbers of total PBS57^+^ iNKT cells and their four distinct stages in development, in WT (open bars), *Ccr4^−/−^* (gray bars), *Ccr7^−/−^* (black bars), and *Ccr4^−/−^*x*Ccr7^−/−^* (shaded bars) mice. (**D**) The percentage and absolute cell numbers of total PBS57^+^ iNKT cells in WT and *Ccr7^−/−^* mice injected with soluble IL-15/IL-15Rα complexes (black bars) or PBS (open bars), along with the absolute numbers of *Ccr7^−/−^* iNKT cell subsets at the indicated developmental stages. (C and D) Error bars represent SEM. Data in (A)–(C) represent a minimum of five mice per group in at least two experimental repeats; data in (D) represent four mice per group in two experimental repeats. An unpaired Student two-tailed *t* test was used: *p < 0.05, **p < 0.01, ***p < 0.001.

Finally, given the requirements for medullary microenvironments during intrathymic iNKT cell development, we performed flow cytometric analysis of thymocytes using PBS57/CD1d tetramers (38), in combination with the iNKT cell maturation markers CD24, CD44 and NK1.1 (39). In contrast to stages in both conventional and Foxp3^+^ regulatory T cell development, whereas intrathymic iNKT cell development involved expression of CCR7, CCR4 was notably absent, with the former being restricted to early developmental stages (Fig. 5A). Moreover, this pattern of chemokine receptor expression correlated with a significant reduction in the proportions and frequencies of iNKT cells in the thymi of *Ccr7^−/−^* but not *Ccr4^−/−^* mice (Fig. 5B), notably stages 1–3 of development (Fig. 5C). Such defects were only slightly enhanced by combined CCR4 deficiency (Fig. 5A–C). Thus, CCR7 is expressed by early iNKT cell subsets and plays a role in their intrathymic development. We hypothesized that the defect in iNKT cell development in the absence of CCR7 may, at least in part, be a consequence of diminished CCR7 dependent thymocyte migration into the thymic medulla, a microenvironment that provides IL15 *trans*-presentation during iNKT cell development (9). To test this, soluble IL-15/IL-15Rα complexes were injected into WT and *Ccr7^−/−^* hosts, and iNKT cell development was examined after 4 d. Fig. 5D shows a significant increase in the frequency of total iNKT cells in *Ccr7^−/−^* hosts receiving IL-15/IL-15Rα compared with PBS injected *Ccr7^−/−^* controls, with numbers of iNKT cells equaling those observed in WT controls receiving PBS. Moreover, IL-15/IL-15Rα treatment of *Ccr7^−/−^* mice selectively increased iNKT cell numbers at stage 3 in their developmental program (Fig. 5D), correlating with the known effects of IL-15 on later stages of iNKT cell development (40). Thus, the reduction in iNKT cells seen in this study in the thymus of *Ccr7^−/−^* mice fits well with the idea that CCR7 enables developing iNKT cells to enter the thymic medulla and gain access to mTEC, known providers of IL-15/IL-15Rα (9).

## Discussion

The intrathymic migration of developing thymocytes through distinct cortical and medullary microenvironments plays an important role in the generation of αβT cells. With regard to the conventional αβT cell pool, CCR7 has been shown to play a prominent role in this process, with *Ccr7^−/−^* mice displaying a reduced capacity for positively selected thymocytes to enter medullary areas of the thymus (10). Interestingly, although the medullary accumulation of SP thymocytes is reduced in *Ccr7^−/−^* mice, small medullary areas are present that contain reduced numbers of SP4 and SP8 thymocytes (10), suggesting the involvement of additional chemokine receptors. With this in mind, and as positive selection is known to induce alterations in the responsiveness of developing thymocytes to multiple chemokines (20), we sought to identify additional chemokine receptors that may be involved in cortex-to-medulla migration. In addition, given that access to the thymic medulla is important during the development of other αβT cell lineages (8, 9), we compared chemokine receptor expression and requirements of Foxp3^+^ nT-Reg and CD1d-restricted iNKT cells with those of conventional αβT cells. We show in this study that transient upregulation of CCR4 maps to a narrow window in the positive selection of both Foxp3^+^ nT-Reg and conventional SP4 αβT cells but not iNKT cells. Thus, CCR4 is expressed at stages of SP4 thymocyte maturation prior to expression of high levels of CCR7 and is absent from more mature CD69^−^ SP4 thymocytes. However, detailed analysis performed here of adult thymus showed *Ccr4^−/−^* thymocytes developed normally, either in the steady state, when in competition with WT thymocytes, or in presence of combined *Ccr7* deficiency. Such findings indicate that the defective Foxp3^+^ nT-Reg (22) and conventional αβT cell development (27) seen in *Aire^−/−^* mice is not because of their reduced levels of the CCR4 ligands CCL17/CCL22. Whether Aire is involved in controlling the intrathymic expression of other chemokines that are required for the normal development and migration of αβT cells is not clear. In addition, the generation and analysis of adult *Ccr4^−/−^*x*Ccr7^−/−^* DKO mice in this study, and the comparable medullary accumulation of SP thymocytes in *Ccr7^−/−^* and *Ccr4^−/−^*x*Ccr7^−/−^* DKO mice rules out the possibility that, in the steady-state adult thymus, CCR4 partially compensates for CCR7 with regard to thymic medulla entry. Although our findings demonstrate that CCR4 is not required for adult intrathymic T cell development and migration, its expression pattern makes it a useful cell surface marker that defines a narrow and early window in the generation of both conventional SP4 thymocytes and Foxp3^+^ nT-Reg.

Although CCR7 has been widely studied in the context of conventional αβT cell development (10, 12, 13, 34, 41), less is known about its possible role in the intrathymic development and migration of other T cell subsets. We found that CCR7 deficiency impacted upon both Foxp3^+^ regulatory and iNKT αβT cell lineages. Specifically, the intrathymic development of iNKT cells was significantly reduced, a finding that correlated with CCR7 expression by early iNKT cell subsets. Given the importance of this chemokine receptor in the intrathymic migration of conventional αβT cells, the finding that iNKT cells are dependent on CCR7 is compatible with their need to enter medullary thymic microenvironments during their development to gain access to *trans*-presentation of IL-15 by mTEC (9). Our findings also collectively highlight CCR7 as a common mechanism for the medullary relocation that occurs during the development of conventional αβT cells (10), γδT cells (15), and now CD1d-restricted iNKT cells. Interestingly, analysis of Foxp3^+^ nT-Reg development showed that absence of CCR7, either individually or in combination with CCR4, did not have an impact on the generation of CD25^+^Foxp3^−^ nT-Reg precursors but did result in an increase in the numbers of CD25^+^Foxp3^+^ nT-Reg in the thymus. Thus, CCR7 plays a role in controlling the balance of intrathymic nT-Reg populations but not during early stages of their development. It is currently unclear why the thymus of *Ccr7^−/−^* mice shows an increase in the CD25^+^Foxp3^+^ nT-Reg population. One possibility is that, as with αβT cells in the neonate (13), CCR7 plays a role in the emigration of Foxp3^+^ nT-Reg from the thymus and that increased numbers reflect their intrathymic accumulation. Such a scenario is perhaps consistent with the previously reported mislocalization of Foxp3^+^ cells in the thymus of *Ccr7^−/−^* mice (16, 17). However, normal numbers of CD25^+^Foxp3^−^ nT-Reg precursors in *Ccr7^−/−^* mice (the generation of which is mTEC dependent) would suggest that developing nT-Reg still have sufficient access to medullary regions in the absence of CCR7. Although this may suggest roles for additional chemokine receptors in early stages of nT-Reg development, our study also rules out a role for CCR4 in this process, despite its restricted expression pattern during positive selection.

In conclusion, our study highlights the differential importance of CCR7 in the intrathymic development of multiple αβT cell lineages, and provides a mechanism by which developing iNKT cells gain access to medullary thymic microenvironments to complete their maturation. Such findings further underline the importance of cortical and medullary thymic compartmentalization during T cell development in the thymus, showing that both innate and adaptive αβT cells share a common chemokine receptor for their intrathymic migration.
